# Racial Disparities in Breastfeeding Initiation and Duration Among U.S. Infants Born in 2015

**DOI:** 10.15585/mmwr.mm6834a3

**Published:** 2019-08-30

**Authors:** Jennifer L. Beauregard, Heather C. Hamner, Jian Chen, Wendy Avila-Rodriguez, Laurie D. Elam-Evans, Cria G. Perrine

**Affiliations:** ^1^Divison of Nutrition, Physical Activity, and Obesity, National Center for Chronic Disease Prevention and Health Promotion, CDC; ^2^Epidemic Intelligence Service, CDC; ^3^Immunization Services Division, National Center for Immunization and Respiratory Diseases, CDC.

Surveillance of U.S. breastfeeding duration and exclusivity has historically reported estimates among all infants, regardless of whether they had initiated breastfeeding. These surveillance estimates have consistently shown that non-Hispanic black (black) infants are less likely to breastfeed, compared with other racial/ethnic groups.* Less is known about disparities in breastfeeding duration when calculated only among infants who had initiated breastfeeding, compared with surveillance estimates based on all infants. CDC analyzed National Immunization Survey-Child (NIS-Child) data for infants born in 2015 to describe breastfeeding duration and exclusivity at ages 3 and 6 months among all black and non-Hispanic white (white) infants, and among only those who had initiated breastfeeding. When calculated among all infants regardless of breastfeeding initiation, breastfeeding differences between black and white infants were 14.7 percentage points (95% confidence interval [CI] = 10.7–18.8) for any breastfeeding at age 3 months and were significantly different for both any and exclusive breastfeeding at both ages 3 and 6 months. Among only infants who had initiated breastfeeding, the magnitude of black-white differences in breastfeeding rates were smaller. This was most notable in rates of any breastfeeding at 3 months, where the percentage point difference between black and white infants was reduced to 1.2 (95% CI = −2.3–4.6) percentage points and was no longer statistically significant. Black-white disparities in breastfeeding duration result, in part, from disparities in initiation. Interventions both to improve breastfeeding initiation and to support continuation among black mothers might help reduce disparities.

Breastfeeding has numerous health benefits for infants and mothers. Breastfed infants have reduced risk for ear, respiratory, and gastrointestinal infections and might be less likely to develop asthma, obesity, and diabetes (*1*). Mothers who breastfeed have a lower risk for developing type 2 diabetes, hypertension, and breast and ovarian cancers (*2*). U.S. breastfeeding surveillance has consistently demonstrated that rates of breastfeeding initiation, duration, and exclusivity are 10–20 percentage points lower among black infants, compared with white infants.^†^

NIS-Child is an ongoing, nationally representative random-digit–dialed telephone survey of U.S. households of children aged 19–35 months. From 2011 to 2017, the NIS-Child used a dual landline and mobile telephone sample frame.^§^ Although NIS-Child primarily assesses childhood vaccination coverage, breastfeeding questions were added in 2001 and are the primary data source for U.S. breastfeeding surveillance. Each cross-sectional survey includes children born in 3 different calendar years; for this analysis of infants born in 2015, data from the 2016–2017 surveys were combined, consistent with national surveillance estimates. Landline sample response rates were 55.7% in 2016 and 51.9% in 2017. Mobile telephone sample response rates were 32.1% in 2016 and 25.0% in 2017. Children’s breastfeeding history and race/ethnicity were reported by their parents or guardians.

Breastfeeding initiation rates were calculated for black and white infants born in 2015. Rates of any breastfeeding and exclusive breastfeeding (defined as only breast milk and no solids, water, or other liquids) at ages 3 and 6 months were calculated for black and white infants using two sets of denominators. The first denominator included all infants of the respective racial/ethnic group regardless of breastfeeding initiation. The second denominator included only infants of the respective racial/ethnic group who had initiated breastfeeding. The absolute percentage point difference in each breastfeeding rate between black and white infants was also estimated (hereafter, black-white difference). Estimates were weighted and accounted for the NIS complex sampling design. Data were analyzed using SAS (version 9.4; SAS Institute) and SUDAAN (version 11.0.3; RTI International).

Black women were more likely than were white women to have incomes <100% of the poverty level (49.3% versus 17.8%), to receive Special Supplemental Nutrition Program for Women, Infants, and Children benefits (78.2% versus 34.1%), and to be unmarried (65.5% versus 23.9%); they also had less education and were younger (Table 1). In 2015, 69.4% of black infants initiated breastfeeding, compared with 85.9% of white infants, a difference of 16.5 percentage points (p<0.05) (Table 2).

**TABLE 1:** Demographic characteristics of non-Hispanic white and non-Hispanic black infants born in 2015 included in national prevalence estimates of breastfeeding initiation and duration at ages 3 and 6 months — National Immunization Survey-Child, United States, 2016–2017*

Characteristic	Non-Hispanic white (n = 9,907)		Non-Hispanic black (n = 1,607)	
	No.	% (95% CI)^†^	No.	% (95% CI)^†^
**% of poverty level^§^**				
<100	1,312	17.8 (16.5–19.1)	635	49.3 (45.5–53.1)
100–199	1,703	18.7 (17.4–20.0)	366	21.0 (18.2–23.8)
200–399	2,909	27.9 (26.5–29.3)	327	16.1 (13.7–18.4)
400–599	1,967	17.7 (16.5–19.0)	110	5.8 (4.3–7.3)
≥600	2,016	17.9 (16.6–19.3)	169	7.8 (5.2–10.4)
**Recipient of WIC** ^¶^				
Yes	2,723	34.1 (32.5–35.8)	1,137	78.2 (75.5–80.9)
No, but eligible	836	9.0 (8.1–9.8)	107	6.8 (5.0–8.5)
Ineligible	6,298	56.9 (55.2–58.6)	356	15.0 (12.8–17.2)
**Mother’s education**				
Less than high school diploma or GED	460	7.4 (6.3–8.4)	199	16.2 (12.7–19.7)
High school diploma or GED	1,394	20.2 (18.8–21.6)	391	32.2 (28.5–35.8)
Some college	2,435	23.4 (22.0–24.8)	491	26.3 (23.2–29.4)
College graduate	5,618	49.1 (47.4–50.7)	526	25.3 (22.3–28.3)
**Mother's age group (yrs)**				
<20	70	1.1 (0.7–1.5)	40	2.8 (1.6–4.0)
20–29	2,943	34.4 (32.8–36.1)	679	45.2 (41.4–49.0)
≥30	6,894	64.5 (62.8–66.1)	888	52.0 (48.2–55.8)
**Mother’s marital status**				
Married	8,097	76.1 (74.6–77.7)	682	34.5 (31.1–37.8)
Unmarried	1,810	23.9 (22.3–25.4)	925	65.5 (62.2–68.9)

**Abbreviations:** GED = general educational development certificate; WIC = Special Supplemental Nutrition Program for Women, Infants, and Children.

* Based on National Immunization Survey-Child data from survey years 2016–2017, among infants born in 2015.

^†^ Statistics in this table are based on participants who responded to questions about any breastfeeding at ages 3 and 6 months (N = 11,514). Sample sizes are slightly smaller for participants who also responded to questions about exclusive breastfeeding at ages 3 and 6 months.

^§^ Ratio of self-reported family income to the poverty threshold value defined by the U.S. Census Bureau.

^¶^ Sample sizes for the proportions of participants receiving WIC are slightly smaller due to missing data on WIC status.

**TABLE 2:** Breastfeeding initiation and duration at ages 3 and 6 months* among non-Hispanic black and non-Hispanic white infants born in 2015 — National Immunization Survey-Child, United States, 2016–2017^†^

Breastfeeding indicator	All infants					Infants who had initiated breastfeeding				
	Non-Hispanic white		Non-Hispanic black		Percentage point difference^§^	Non-Hispanic white		Non-Hispanic black		Percentage point difference^§^
	No.	% (95% CI)	No.	% (95% CI)	% (95% CI)	No.	% (95% CI)	No.	% (95% CI)	% (95% CI)
Initiated breastfeeding	9,907	85.9 (84.7 to 87.1)	1,607	69.4 (65.9 to 73.0)	16.5 (12.7 to 20.2)^¶^	8,729	N/A	1,159	N/A	N/A
Any breastfeeding at age 3 mos	9,907	72.7 (71.2 to 74.2)	1,607	58.0 (54.2 to 61.7)	14.7 (10.7 to 18.8)^¶^	8,729	84.7 (83.4 to 85.9)	1,159	83.5 (80.3 to 86.7)	1.2 (−2.3 to 4.6)
Exclusive breastfeeding through age 3 mos	9,537	53.0 (51.4 to 54.7)	1,573	36.0 (32.2 to 39.7)	17.0 (12.9 to 21.2)^¶^	8,359	62.2 (60.5 to 63.9)	1,125	52.3 (47.8 to 56.9)	9.9 (5.0 to 14.7)^¶^
Any breastfeeding at age 6 mos	9,907	62.0 (60.4 to 63.6)	1,607	44.7 (40.9 to 48.5)	17.3 (13.1 to 21.4)^¶^	8,729	72.2 (70.6 to 73.8)	1,159	64.4 (60.2 to 68.6)	7.8 (3.3 to 12.3)^¶^
Exclusive breastfeeding through age 6 mos	9,537	29.5 (28.0 to 31.1)	1,573	17.2 (14.1 to 20.2)	12.4 (8.9 to 15.8)^¶^	8,359	34.7 (32.9 to 36.4)	1,125	25.0 (20.8 to 29.2)	9.7 (5.1 to 14.2)^¶^

**Abbreviations:** CI = confidence interval; N/A = not applicable.

* Breastfeeding initiation was determined according to participant's response to the question “Was [child] ever breastfed or fed breast milk?” Breastfeeding duration was determined according to participant's response to the question “How old was [child’s name] when [child’s name] completely stopped breastfeeding or being fed breast milk?” Exclusive breastfeeding was defined as only breast milk (no solids, no water, and no other liquids). To assess the duration of exclusive breastfeeding, participants were asked two questions about age: 1) “How old was [child’s name] when he/she was first fed formula?” and 2) “How old was [child’s name] when he/she was first fed anything other than breast milk or formula?” (This includes juice, cow’s milk, sugar water, baby food, or anything else that [child] might have been given, even water).

^†^ Based on National Immunization Survey-Child data from survey years 2016–2017, among infants born in 2015.

^§^ Differences in breastfeeding rates between non-Hispanic black and non-Hispanic white infants.

^¶^ Differences in breastfeeding rates between non-Hispanic black and non-Hispanic white infants are statistically significant (p<0.05, two-sample test of proportions).

Among all infants, black infants had a significantly lower rate of any breastfeeding at age 3 months (58.0%) than did white infants (72.7%); at age 6 months, the rates were 44.7% among black infants and 62.0% among white infants (p<0.05). Rates for exclusive breastfeeding at age 3 months were 36.0% among black infants and 53.0% among white infants; at age 6 months, the rates were 17.2% among black infants and 29.5% among white infants (p<0.05) (Table 2). At age 3 months, black-white differences were 14.7 percentage points for any breastfeeding (95% CI = 10.7–18.8) and 17.0 percentage points for exclusive breastfeeding (95% CI = 12.9–21.2). At age 6 months, black-white differences were 17.3 percentage points for any breastfeeding (95% CI = 13.1–21.4) and 12.4 percentage points for exclusive breastfeeding (95% CI = 8.9–15.8) (Table 2).

Among only infants who had initiated breastfeeding, the magnitude of black-white differences in any and exclusive breastfeeding rates were smaller (Table 2). This was most notable in rates of any breastfeeding at 3 months, where the percentage point difference between black and white infants was reduced from 14.7 (95% CI = 10.7–18.8) to 1.2 (95% CI = −2.3–4.6) percentage points; this difference was no longer statistically significant. The black-white difference in exclusive breastfeeding at age 3 months was reduced from 17.0 percentage points (95% CI = 12.9–21.2) to 9.9 percentage points (95% CI = 5.0–14.7), in any breastfeeding at 6 months from 17.3 percentage points (95% CI = 13.1–21.4) to 7.8 percentage points (95% CI = 3.3–12.3), and in exclusive breastfeeding at age 6 months from 12.4 percentage points (8.9–15.8) to 9.7 percentage points (95% CI = 5.1–14.2).

## Discussion

Surveillance of U.S. breastfeeding duration and exclusivity, including monitoring for Healthy People 2020^¶^ objectives, reports estimates among all infants, regardless of whether they had initiated breastfeeding. The findings in this report demonstrate that differences between black and white infants in any and exclusive breastfeeding at ages 3 and 6 months are caused, in part, by racial/ethnic differences in breastfeeding initiation. Interventions to improve breastfeeding initiation and support continuation among black mothers might be important to closing the black-white gap in duration.

Black mothers disproportionately experience a number of barriers to breastfeeding, including lack of knowledge about breastfeeding; lack of peer, family, and social support; insufficient education and support from health care settings; and concerns about navigating breastfeeding and employment (*3*). Subjective norms, or perceptions of approval from others who are important to the person (e.g., family members), are important drivers of breastfeeding behaviors, particularly among black women (*3*). Increasing interpersonal support for breastfeeding might help increase breastfeeding initiation and duration among black women, who might lack breastfeeding role models in their social networks and be more likely to face negative perceptions of breastfeeding among their peers and communities (*3*,*4*). For example, peer counseling might increase breastfeeding initiation and duration among black mothers (*3*).

In the United States, the rate of implementation of evidence-based maternity care practices supportive of breastfeeding is lower among maternity care facilities in neighborhoods with larger black populations (*5*). Hospitals’ use of such practices, which include helping women initiate breastfeeding within the first hour of birth and not providing breastfeeding infants with infant formula without a medical indication, increases rates of breastfeeding initiation, duration, and exclusivity (*6*). A recent analysis indicated that making improvements in these practices among maternity care facilities in four southern states reduced black-white disparities in breastfeeding initiation (*7*).

Returning to work is another major barrier to breastfeeding initiation and continuation, particularly for black women (*3*). A woman’s plans for returning to work are associated with her intention to breastfeed; specifically, women planning to return to work before 12 weeks postpartum, planning to work full-time, or both were less likely to intend to exclusively breastfeed, compared with women planning to return to work after 12 weeks postpartum, planning to work part-time, or both (*8*). Black women, especially those with a low income, return to work earlier than do women in other racial/ethnic groups and are more likely to experience challenges to breastfeeding or expressing milk, including inflexible work hours (*9*). Policies that enable taking paid leave after giving birth, flexible work schedules, and support for breastfeeding or expressing milk at work might help improve breastfeeding intention, initiation, and duration.**

The findings in this report are subject to at least three limitations. First, response rates averaged 53.8% for the landline sample and 28.6% for the mobile telephone sample; further, households without a telephone are not represented. The possibility exists that selection bias occurs even after adjusting weights for nonresponse and noncoverage. Second, maternal reports of breastfeeding behaviors could be subject to recall bias because mothers reported these behaviors when their children were aged 19–35 months and to social desirability bias because of a desire to provide socially acceptable responses. However, maternal recall of breastfeeding behavior has been found to be valid and reliable, especially when recalled within 3 years (*10*). Finally, although this report focuses only on black-white breastfeeding differences, lower rates of breastfeeding duration and exclusivity among Hispanic infants, compared with non-Hispanic white infants, have been documented (*3*). However, because Hispanic and white infants have similar rates of breastfeeding initiation, the methods applied in this report did not affect estimates of breastfeeding duration and exclusivity.

Breastfeeding provides optimal nutrition to infants and provides health benefits for both infants and mothers, and CDC works to increase breastfeeding rates among all mothers in the United States. In order to address disparities in breastfeeding duration, continued efforts are needed to increase rates of breastfeeding initiation and support continuation of breastfeeding among black women. Closing the black-white gap in breastfeeding duration might require efforts of multiple groups. Families, hospitals, and employers can help black women initiate and continue breastfeeding, thereby providing their infants with optimal nutrition.

**Summary**

**What is already known on this topic?**

Rates of breastfeeding duration and exclusivity, calculated for all infants regardless of whether they had initiated breastfeeding, are lower among black infants than among white infants.

**What is added by this report?**

Among infants who had initiated breastfeeding, differences between black infants and white infants in any and exclusive breastfeeding at ages 3 and 6 months were smaller but still present.

**What are the implications for public health practice?**

Increasing rates of breastfeeding initiation and supporting continuation of breastfeeding among black women might help reduce disparities in breastfeeding duration. Strategies might include improving peer and family support, access to evidence-based maternity care, and employment support.

## Footnotes

* https://www.cdc.gov/breastfeeding/data/nis_data/rates-any-exclusive-bf-socio-dem-2015.htm.

† https://www.cdc.gov/breastfeeding/data/nis_data/index.htm.

§ https://www.cdc.gov/breastfeeding/data/nis_data/methods.html.

¶ https://www.healthypeople.gov/2020/topics-objectives/topic/maternal-infant-and-child-health/objectives.

** https://www.cdc.gov/breastfeeding/resources/calltoaction.htm.
